# Ten-year survival of women with invasive breast cancer by stage, molecular subtype, and grade combined: a population-based study

**DOI:** 10.1093/oncolo/oyag338

**Published:** 2026-08-25

**Authors:** Fabiola Giudici, Diego Serraino, Fabio Puglisi, Samuele Massarut, Lorenzo Gerratana, Serena Scomersi, Federica Toffolutti, Ettore Bidoli, Luigino Dal Maso, Friuli Venezia Giulia Cancer Registry working group, Tiziana Angelin, Emilia De Santis, Ornella Forgiarini, Elodie Lazzaretto, Mariella Losciale, Martina Taborelli

**Affiliations:** Cancer Epidemiology Unit, Centro di Riferimento Oncologico di Aviano (CRO) IRCCS, Aviano, 33081, Italy; Cancer Epidemiology Unit, Centro di Riferimento Oncologico di Aviano (CRO) IRCCS, Aviano, 33081, Italy; Department of Medical Oncology, Centro di Riferimento Oncologico di Aviano (CRO) IRCCS, Aviano, 33081, Italy; Department of Medicine, University of Udine, Udine, 33100, Italy; Breast Surgery Unit, Centro di Riferimento Oncologico di Aviano (CRO) IRCCS, Aviano, 33081, Italy; Department of Medical Oncology, Centro di Riferimento Oncologico di Aviano (CRO) IRCCS, Aviano, 33081, Italy; Department of Medicine, University of Udine, Udine, 33100, Italy; Breast Surgery Unit, Cattinara University Hospital, Trieste, 34149, Italy; Cancer Epidemiology Unit, Centro di Riferimento Oncologico di Aviano (CRO) IRCCS, Aviano, 33081, Italy; Cancer Epidemiology Unit, Centro di Riferimento Oncologico di Aviano (CRO) IRCCS, Aviano, 33081, Italy; Cancer Epidemiology Unit, Centro di Riferimento Oncologico di Aviano (CRO) IRCCS, Aviano, 33081, Italy

**Keywords:** breast cancer, 10-year net survival, population-based cancer registries, molecular profile cancer, stage

## Abstract

**Background:**

Stage at diagnosis, histologic grade, and molecular subtypes are established prognostic indicators for women with breast cancer (BC), but their impact on population-based survival estimates is poorly documented. This study assessed long-term BC survival trends according to these factors.

**Methods:**

We identified women aged <75 years diagnosed with stage I-IV invasive BC between 2004 and 2014 from the Friuli Venezia Giulia (north-eastern Italy) Cancer Registry (*N* = 10 467). Follow-up through 2023 was used to estimate overall and net survival (NS) according to combinations of prognostic factors.

**Results:**

The highest 10-year NS (10-NS) was observed in women with stage I HR+/HER2− subtype (98.8%), while it was 35.1% for stage III triple-negative (TN) subtype. Among women with stage IV BC, 10-NS was 18.9% for those HR−/HER2+, 11.5% for those HR+/HER2+, 8.3% for HR+/HER2−, and 6.7% for TN. For each stage, NS decreased with increasing grade. Comparing periods of diagnosis (2004-2009 vs 2010-2014), 10-NS remained >90% for women with stage I BC, but improved for stage III and aggressive subtypes (HR−/HER2+ and TN). In women with stage III HR−/HER2+ BC, 10-NS increased from 48.6% to 87.9% (+39 percentage points), while when diagnosis was stage III TN BC it rose from 28.8% to 42.3%

**Conclusion:**

Population-based estimates of long-term BC survival by combined stage, grade, and molecular subtype can inform the interpretation of evolving therapeutic strategies and improve risk stratification for patient follow-up.

Implication for practice This population-based study provides estimates of long-term survival for Italian women with breast cancer by stage at diagnosis, molecular subtype, and histologic grade jointly. The combined impact of pathological factors (stage, grade) and immunohistochemical features (molecular subtype) on 10-year survival allows for an accurate risk stratification of patients, one of the most important aspects for designing and implementing personalized care models. This information can help define tailored follow-up plans, improve women’s quality of life and conduct stratified cost analyses.

## Introduction

Breast cancer (BC) is the most commonly diagnosed cancer worldwide,^1^ and its burden has been rising over the past decades, particularly in women under 50 years of age.^2^ In Italy and other European countries, more than a quarter (ie, 30%) of new cancer cases diagnosed in women in 2024 were BC,^3^ affecting, approximately 53 000 Italian women, with 15 500 deaths.^4^ Moreover, it was estimated that one million women will live in Italy in 2030^5^ and 5.5 million (in 2020) in Europe^6^ after a BC diagnosis.

It is acknowledged that BC is a heterogeneous disease with a wide spectrum of clinical, pathologic, and molecular features.^7^ Although BC classification continues to evolve with the discovery of new biomarkers and a deeper understanding of their role in tumor progression, in routine clinical practice, BC is still subdivided into four clinical subgroups based on the immunohistochemical expression of hormone receptors (HR: estrogen receptor [ER], progesterone receptor [PR]) and human epidermal growth factor receptor 2 (HER2).

Four main groups can be defined according to receptor status: HER2−/HR+, HER2+/HR+, HER2+/HR− and HER2−/HR− (referred to as TN) groups.^8^ Determination of HER2 status is usually based on immunohistochemistry, but in equivocal cases requires confirmation by assessing *HER2/neu* gene amplification using in situ hybridization.^9^ The different molecular subtypes of BC have distinct biological features, including variability in recurrence and tendency to develop metastases,^10–12^ as well as response patterns to treatments,^13^ which have been shown to influence survival.^14^

The prognostic assessment of patients with BC relies on a set of widely validated factors, including tumor stage at diagnosis^15^^,^^16^ molecular subtype,^17^ and histologic grade^18^ which have guided treatment decisions over the past decade.^19^^,^^20^ However, such information from population-based cancer registries is still limited.^21^^,^^22^ In particular, few registries collect ER, PR and HER2 data,^23^ the key distinguishing markers for molecular subtypes of breast cancer. In addition, the impact of stage and molecular subtypes is rarely assessed separately, with the most recent statistics based on population-based data only reporting net survival (adjusted for patient life expectancy) at 5 years after diagnosis for patients with BC overall.^24–26^ Only a few studies have estimated long-term survival,^27^^,^^28^ while information on survival beyond 5 years after diagnosis according to the stage at diagnosis, tumor grade, and/or molecular profile is scant in Italy^29^^,^^30^ and elsewhere.^31^^,^^32^

The present study aims to evaluate the combined impact of major prognostic factors on BC survival in Italy, up to 10 years after diagnosis.

## Methods

### Cases included

All first primary cases of invasive female BC (International Classification of Diseases for Oncology (ICD-10)^33^ sites C50.0-C50.9) diagnosed between 1 January 2004 and 31 December 2014, with follow-up until 31 December 2023, were retrieved from the Friuli Venezia Giulia Cancer Registry in north-eastern Italy, covering a population of 1.2 million inhabitants (0.61 million women). Case registration follows the guidelines of the International Agency for Research on Cancer^34^ and the recommendations of the European Network of Cancer Registries.

**Figure 1.**
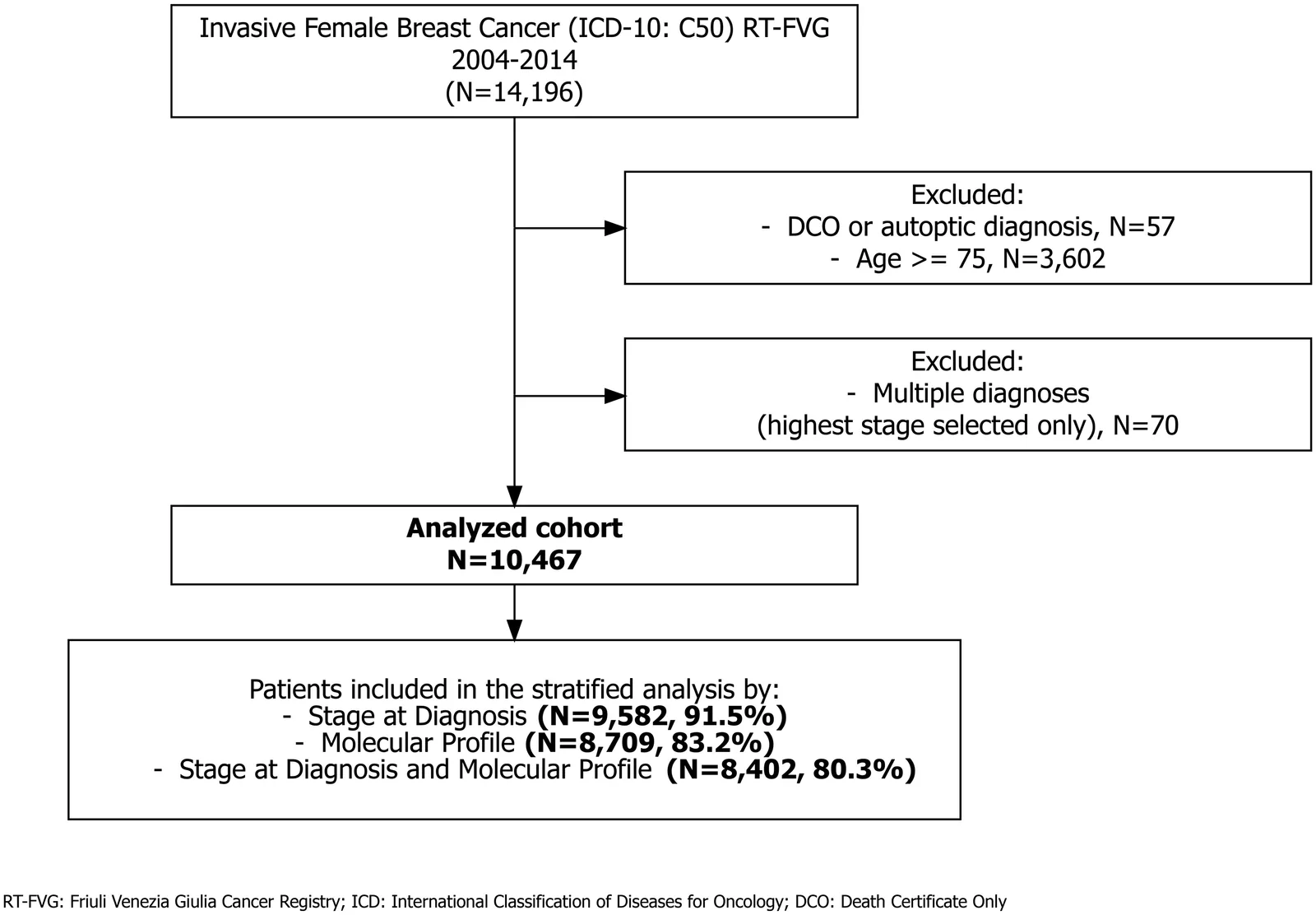
Flowchart of patient selection.

Among the identified patients with BC (*n* = 14 196), women with the following characteristics were excluded from the cohort: BC diagnosis by autopsy or death certificate only (*n* = 57). Patients older than 74 years (*n* = 3602) were excluded because estimates of long-term NS are less reliable for older age groups.^35–36^ If women had two breast cancer diagnoses in the same incidence period, the higher stage of diagnosis was considered in the analysis (*n* = 70). The cohort enrolled included 10 467 women (Figure 1).

TNM (7th edition) was used to identify the stage at diagnosis, available for 91.5% of the cases (*n* = 9582). Tumors were classified as ER negative (ER−) or PR negative if < 10% ER or PR expression. HER2 expression was routinely assessed with immunohistochemistry (IHC) and verified with in situ hybridization if the IHC results were borderline.^9^ Four IHC subtypes were analyzed HR+/HER2−, HR+/HER2+, HR−/HER2+, and HR−/HER2 − (triple-negative breast cancer, TN). In total, *n* = 8709 women had a known IHC subtype, ie, 83.2% of the whole cohort (Figure 1).

### Statistical methods

Given the descriptive nature of this population-based study, which includes all eligible women with invasive BC enrolled in the registry during the study period, calculations regarding sample size and statistical power were not applied. Survival time was measured from the patient’s date of diagnosis until death or 31 December 2023, whichever came first. We estimated the 5- and 10-year net survival (NS) for patients according to several prognostic factors. NS is the probability that cancer patients survive their cancer up to a given time since diagnosis, after controlling for competing causes of death, using the cohort method and the Pohar Perme approach^35^ that has been proved to produce an unbiased estimate of the NS and represents the “standard” approach to estimate “cause-specific” survival for population-based studies.^15^^,^^25^ Net survival measures the probability of surviving cancer itself, after removing the influence of other causes of death (background mortality). Rather than relying on cause-of-death information, which is susceptible to error and is not recorded consistently enough for reliable international comparisons, net survival adjusts for background mortality using life tables.^36–38^ A survival analysis that accounts for changes in background mortality allows for a more realistic assessment of changes in cause-specific survival over a long period.

Expected survival was computed from the regional life tables provided by the Italian National Institute of Statistics for the CR area, stratified by age (in years), sex, and calendar year.^39^ Patients with unspecified status due to emigration were censored on the day of emigration. All statistical analyses were performed using R version 4.2.3.^40^ The estimation of the net survival through the Pohar-Perme estimator was done using *relsurv* package.^41^ For reproducibility, the R code used to estimate net survival is available in the Supplementary Material. Please note that to reproduce or extend these analyses to other geographic areas, the reference population life tables must be adapted to the specific country or region of interest.

## Results

A total of 10 467 women with invasive BC cases were diagnosed below age 75 years and reported to the Friuli Venezia Giulia Cancer Registry during 2004-2014. Population characteristics and survival at 5 and 10 years are presented in Table 1. More than half of BC cases were diagnosed at age 50-69 years (59.4%), had stage I (50.1%) and grade G2 (53.2%). The most common histotype was infiltrating ductal BC (78.8%), and the most prevalent molecular subtype was HR+/HER2 − (75.0%). The combined distribution of stage at diagnosis and molecular profile is provided in Table S2.

**Table 1.** Patient’s characteristics, overall and net survival at 5 and 10 years from diagnosis. Friuli Venezia Giulia Cancer Registry, 2004-2014, age 20-74 years.

		5-years		10-years	
Characteristic	*N* = 10 467	OS	NS	OS	NS
All women		88.9%	91.5%	79.6%	85.8%
**Age**					
**≤39**	536 (5.1%)	88.3%	88.5%	80.1%	80.8%
** 40-49**	2105 (20.1%)	92.8%	93.4%	88.0%	89.5%
** 50-59**	2586 (24.7%)	90.2%	91.4%	84.1%	87.3%
** 60-69**	3631 (34.7%)	89.0%	92.3%	78.8%	86.7%
** 70-74**	1609 (15.4%)	82.0%	88.1%	63.0%	77.9%
**Period**					
** 2004-2009**	5632 (53.8%)	88.6%	91.0%	79.1%	84.9%
** 2010-2014**	4835 (46.2%)	89.4%	92.0%	80.3%	86.8%
**Histologic grade**					
** G1**	1337 (14.3%)	96.4%	99.2%	90.5%	98.0%
** G2**	5022 (53.7%)	92.8%	95.6%	83.0%	89.8%
** G3**	2998 (32.0%)	83.4%	85.6%	73.7%	78.8%
** Total**	9357				
** Unknown**	1110 (10.6%)				
**Morphology**					
** Adenocarcinomas**	319 (3.0%)	90.2%	93.1%	85.0%	92.4%
**Cystic/mucinous/serous**	190 (1.8%)	91.5%	94.7%	82.3%	91.6%
** Ductal neoplasm**	8246 (78.8%)	89.6%	92.0%	80.2%	86.6%
** Epithelial neoplasms**	231 (2.2%)	76.4%	78.6%	68.4%	73.5%
** Lobular neoplasm**	1268 (12.1%)	89.1%	91.8%	77.3%	83.9%
** Other**	213 (2.0%)	74.1%	76.6%	70.7%	75.0%
**pT**					
** T1**	6507 (69.4%)	95.2%	97.9%	88.4%	95.2%
** T2**	2320 (24.8%)	86.1%	88.6%	72.8%	78.7%
** T3**	212 (2.3%)	67.3%	69.2%	43.4%	46.5%
** T4**	333 (3.6%)	54.9%	56.8%	36.0%	39.5%
** Total**	9372				
** Unknown**	1095 (10.5%)				
**pN**					
** N0**	5505 (60.6%)	95.2%	98.1%	88.7%	96.0%
** N1**	2492 (27.4%)	90.8%	93.1%	80.8%	86.5%
** N2**	653 (7.2%)	82.8%	85.0%	64.7%	69.1%
** N3**	441 (4.9%)	67.2%	69.1%	44.6%	48.7%
** Total**	9091				
** Unknown**	1376 (13.1%)				
**Stage**					
** I**	4821 (50.3%)	96.3%	99.1%	90.2%	97.5%
** II**	3116 (32.5%)	91.8%	94.3%	82.4%	88.5%
** III**	1222 (12.8%)	78.8%	81.1%	58.4%	62.9%
** IV**	423 (4.4%)	21.8%	22.4%	8.4%	9.1%
** Total**	9582				
** Unknown**	885 (9.2%)				
**Molecular profile**					
** HR+/HER2−**	6536 (75.0%)	92.0%	94.7%	82.3%	89.0%
** HR+/HER2+**	872 (10.0%)	88.8%	90.9%	78.7%	83.6%
** HR−/HER2+**	478 (5.5%)	81.9%	83.8%	74.0%	78.8%
** HR−/HER2−**	823 (9.4%)	74.7%	76.6%	68.5%	73.2%
** Total**	8709				
** Unknown**	1758 (16.8%)				

Abbreviations: OS: Overall Survival; NS: Net survival.

The overall 10-year observed survival and NS for the whole cohort were 79.6% and 85.8% (Figure S1).

Women with stage I or low-grade (G1) BC had the most favorable prognosis with 10-year NS exceeding 97% (97.5% and 98.0%, respectively), which became 99.7% when the two prognostic factors were combined (Figure S2). For each stage, NS decreased with increasing grade: the differences in survival between G1 and G3 were 4% in stage I, 11% in stage II and 21% in stage III (Figure S2). Among women with non-metastatic BC, the lowest 10-year NS was found in those with TN and stage III BC (73.2% and 62.9%, respectively) (Table 1).

**Figure 2.**
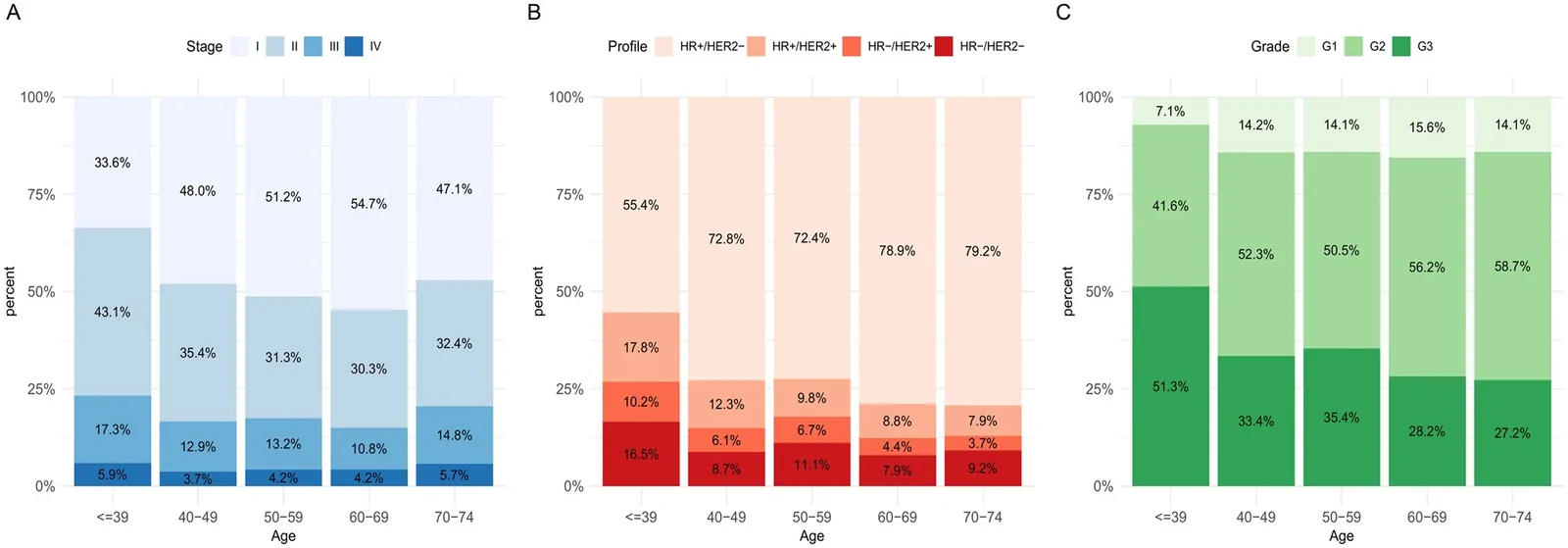
TNM Stage, Molecular profile and Grade according to age at diagnosis for women with invasive breast cancer, Friuli Venezia Giulia Cancer Registry, 2004-2014, age 20-74 years.

Stage, molecular profile and grade distributions varied by age (Figure 2, Table S1). Among patients aged less than 40 years at diagnosis, 33.6% had stage I disease as compared with about 50% for those diagnosed in the other age groups. We observed a higher proportion of the HR−/HER2− (16.5%) and HER2 positive (28.0%) subtypes and G3 BC (51.3%) among younger women. This aggressive pattern is documented by the outcomes of 10-year NS (Table 1, Figure S3A), which remained over 87% for the patients aged 40-69 years, while reached only the 80.8% for the youngest group, similar to that observed in the oldest women (70-74 years; 10-year NS: 77.9%).

We also analyzed the NS for women in each molecular subtype according to age. In women with the HR+/HER2− subtype, patients younger than 40 years showed lower survival, similar to that of patients aged 70-74 years (around 82%), whereas the other age groups had 10-year survivals of around 90%. (Figure S3B). However, in the TN, patients aged 70-74 years showed poor survival (64%), while the remaining age groups showed similar survival (range 72%-76%) (Figure S3B).

**Figure 3.**
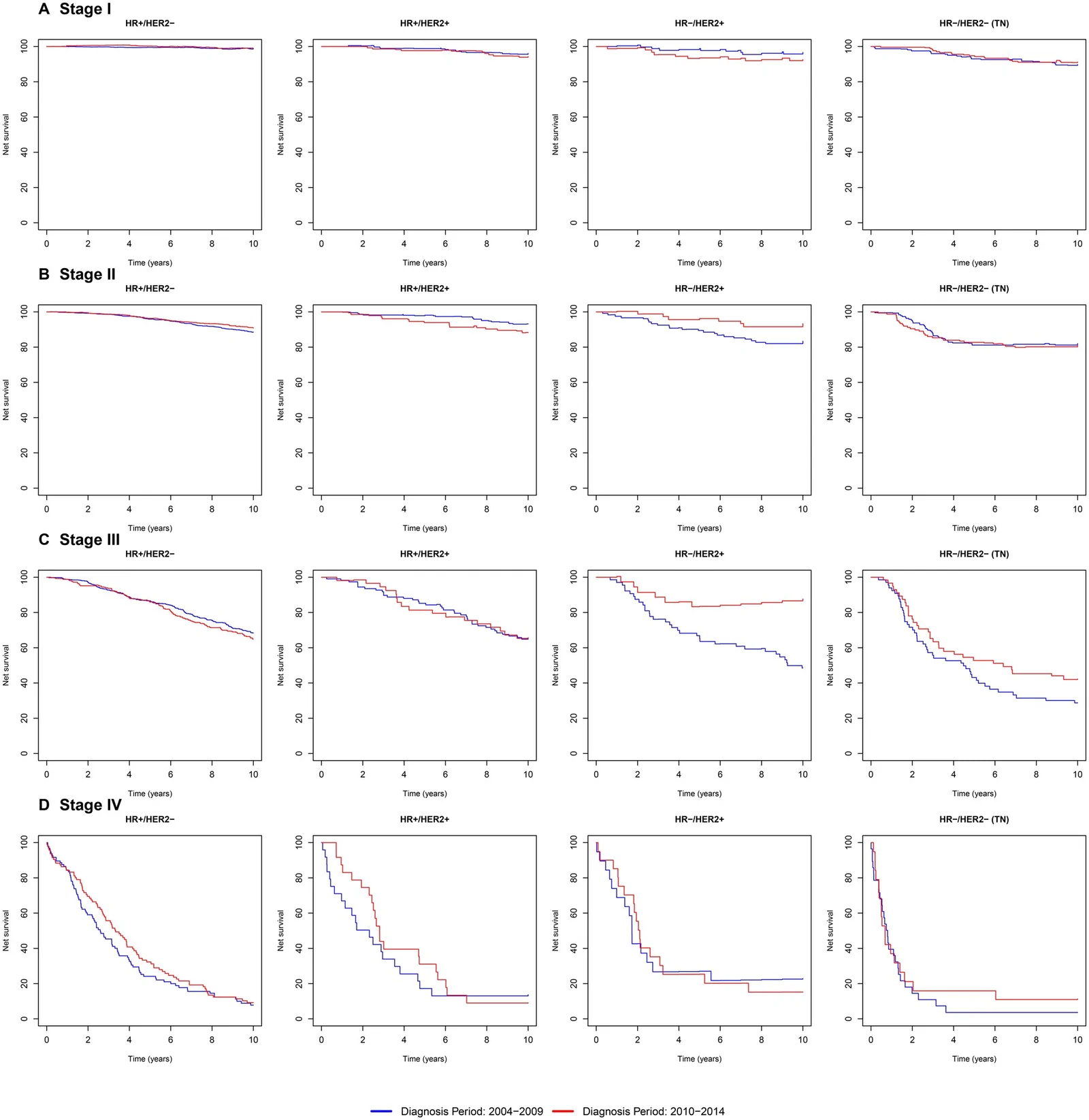
Net Survival (NS) according to TNM Stage, Molecular profile and period of diagnosis, for women with invasive breast cancer, Friuli Venezia Giulia Cancer Registry, 2004-2014, age 20-74 years.

Although both stage and molecular subtypes affected survival (Figure S4A-4B), differences by stage at diagnosis were particularly pronounced (Table 2, Figure 3).

**Table 2.** Overall survival (OS) and Net Survival (NS) according to TNM Stage and Molecular profile, for women with invasive breast cancer, Friuli Venezia Giulia Cancer Registry, 2004-2014, age 20-74 years.

					5-years		10-years	
Stage	Molecular profile	Patients, *N* (%)	Deaths at 10 years, *N*	Deaths at 15 years, *N*	OS	NS	OS	NS
**I (*N* = 4223)**	HR+/HER2−	3407 (81%)	295	477	97.1%	100%	91.2%	98.8%
	HR+/HER2+	353 (8.4%)	35	59	96.6%	98.5%	89.9%	95.5%
	HR−/HER2+	156 (3.7%)	17	26	94.2%	96.2%	89.0%	94.9%
	HR−/HER2−	307 (7.3%)	48	60	91.5%	93.9%	84.2%	90.6%
**II (*N* = 2761)**	HR+/HER2−	1998 (72.4%)	338	500	93.4%	96.1%	82.9%	89.5%
	HR+/HER2+	292 (10.6%)	40	58	94.5%	96.7%	86.2%	91.7%
	HR−/HER2+	160 (5.8%)	28	33	89.8%	91.8%	82.0%	86.9%
	HR−/HER2−	311 (11.3%)	72	89	80.1%	81.9%	76.8%	81.8%
**III (*N* = 1098)**	HR+/HER2−	747 (68.0%)	282	361	83.9%	86.4%	61.9%	66.9%
	HR+/HER2+	142 (12.9%)	54	58	82.3%	84.1%	61.3%	65.2%
	HR−/HER2+	93 (8.5%)	39	44	71.0%	72.5%	57.7%	61.9%
	HR−/HER2−	116 (10.7%)	77	82	46.0%	47.6%	32.5%	35.1%
**IV (*N* = 320)**	HR+/HER2−	187 (58.4%)	172	173	27.3%	28.2%	7.8%	8.3%
	HR+/HER2+	47 (14.7%)	42	43	23.4%	24.0%	10.6%	11.5%
	HR−/HER2+	39 (12.2%)	32	33	25.6%	25.9%	17.9%	18.9%
	HR−/HER2−	47 (14.7%)	44	44	8.5%	8.6%	6.4%	6.7%

Abbreviations: OS: Overall survival (using the Kaplan-Meier method); NS: net survival (using the Pohar-Perme estimator).

**Figure 4.**
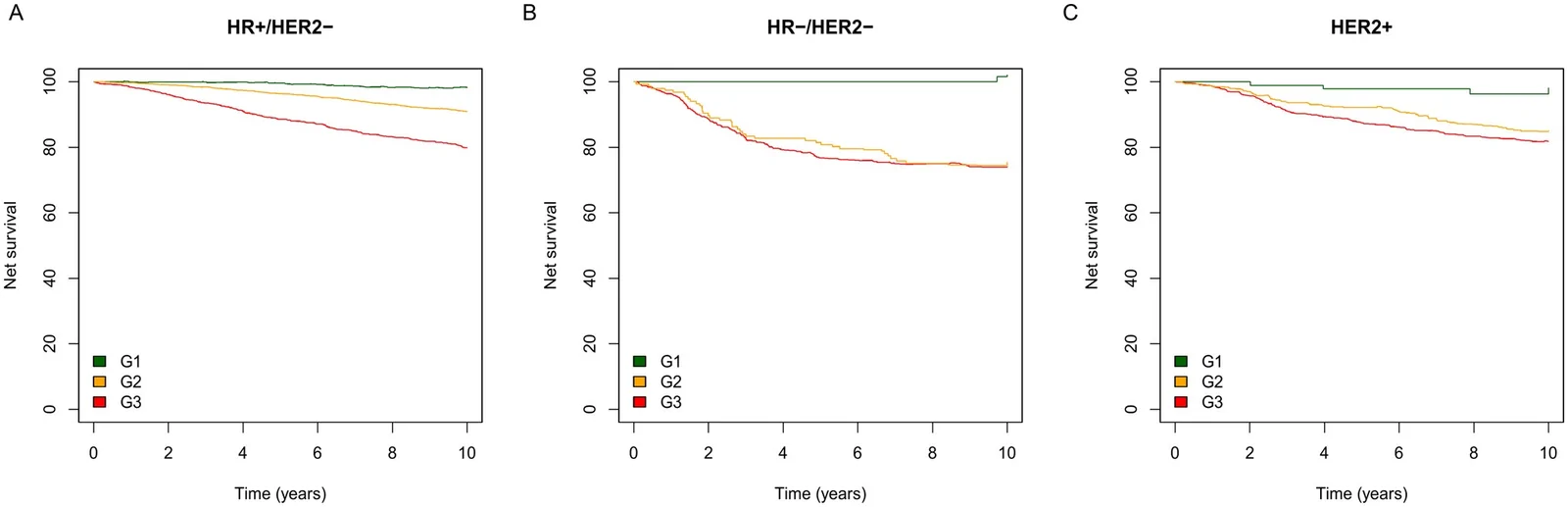
Net Survival (NS) according to HR+/HER2−, HR−/HER2−, HER2+ and Grade for women with invasive breast cancer, Friuli Venezia Giulia Cancer Registry, 2004-2014, age 20-74 years.

For the women with TN subtype, the 10-year NS was 90.6% among those with stage I disease, 81.8% for stage II, 35.1% for stage III BC, and 6.7% among those with stage IV disease (Table 2). Survival was similar between the women with HR−/HER2+ and HR+/HER2+ subtypes when diagnosis was at stage I and II, but not at stage IV disease, where survival was higher for HR−/HER2+ than HR+/HER2+ subtypes (18.9% vs 11.5%). Among women with de novo metastatic BC, those with HR+/HER2− BC had a low 10-NS (8.3%), similar to that of TN (6.7%). In contrast, when this HR+/HER2− disease was diagnosed at stage I, almost all patients were alive 10 years after diagnosis (10-y NS: 98.9%) (Table 2). The combined effect of grade and molecular profile was also investigated: among women with HR+/HER2− BC the prognosis worsened as the grade increased, from a 10-year survival rate of 98.3% for G1 tumors to 79.9% for G3 tumors, the latter survival being similar to that of triple-negative G2 and G3 cancers (respectively, 75.5% and 74.1%) (Figure 4 and Table S4).

Comparing the 2004-2009 and 2010-2014 periods, the NS remained stable for women diagnosed at stage I BC (all subtypes), while it improved in particular for stage III and more aggressive molecular subtypes (HR−/HER2+ and TN): the 10-year NS for women with stage III HR−/HER2+ had an increase of 39 percentage points, from 48.6% to 87.9%. Similarly, for women with stage III TN disease there was an improvement in survival, from 28.8% in the 2004-2009 period to 42.3% in the more recent period (Table S3). For women with stage IV disease, as there are very few women at risk, the results should therefore be interpreted with caution: while we observed an improvement of NS among TN (from 3.7% to 11.4%), there seemed to be a worsening of prognosis for HER2+ women during 2009-2014 more pronounced for HR−/HER2 + (from 22.9% to 15.2%) (Figure 3 and Table S3).

## Discussion

This is the first population-based study in Italy to assess long-term survival of women with BC according to the combinations of stage, molecular subtypes and grade. We confirmed the strong prognostic impact of stage, with 10-year survival exceeding 90% for women with stage I disease (50% of our cohort), regardless of molecular subtype. Within stage III cases, patients with HR + tumors have better outcomes, with a 10-year NS of approximately 60%. In contrast, patients with HR– subtypes, particularly triple-negative BC, showed markedly poorer outcomes, with 10-year NS dropping to 35%. Among women with stage IV disease, those with HR−/HER2+ tumors appeared to have better long-term survival than women with other molecular subtypes.

Although a modest overall increase in 10-year NS was observed between 2004-2009 and 2010-2014 (from 85.3% to 87.2%), significant improvements were seen for patients with advanced-stage disease and more aggressive molecular subtypes. Notably, survival of women with stage III HR−/HER2+ BC increased by approximately 39%, and by 14% for those with stage III TN. Consistent with previous findings,^42^ TN, HER2+ and G3 tumors were more commonly diagnosed among younger women, whereas the proportions of HR + subtypes increased with age. Patients aged ≤39 years experienced poorer survival compared to those aged 40-69 years, largely due to a higher frequency of aggressive tumor biology advanced-stage diagnoses (stage III) and poorly differentiated (G3) cancers, as also reported by other studies.^43^^,^^44^

Few population-based studies have assessed long-term survival according to the combined effect of stage and molecular subtype. A study^27^ by Howlader et al, based on data from the United States Surveillance, Epidemiology and End Results cancer registry, stratified survival by both molecular subtype and stage, but was restricted to only four years of follow-up. Waks et al^45^ focused specifically on stage I HER2+ BC, showing a favorable prognosis for this subgroup, with 5-year survival rates exceeding 92%, regardless of chemotherapy administration. Johansson et al,^28^ using data from a large nationwide Norwegian cancer registry, showed that tumor size, nodal status, grade and molecular subtype are all independent predictors of BC mortality and should be assessed together to estimate prognosis. Their findings aligned with those of our study, particularly regarding excellent long-term outcomes for women with small, node-negative tumors (stage I), with 10-year survival rates exceeding 90% across all molecular subtypes. It should be further noted that histologic grade was an important determinant of prognosis that also allowed risk stratification within a given tumor stage. The proportion of high-grade cases increased with advancing stage: therefore, poorly differentiated tumors, even at an early stage, could have an impact on survival.

A recent comprehensive review of survival analyses based on cancer registry data^46^ reported results consistent with ours, highlighting overall improvements in survival over time, with the most marked gains observed among women with regional disease (ie, Stage III). However, the study reported relative survival only at 5 years and did not stratify the analysis by both stage and molecular subtype.

In our cohort of HR+/HER2– breast cancer patients, histologic grade was a strong determinant of long-term outcomes, with 10-year survival rates exceeding 98% for patients with grade 1 tumors and falling to approximately 80% for grade 3 disease. While contemporary prognostic models often prioritize tumor size, nodal status, and genomic signatures, our findings suggest that histologic grade retains independent and clinically relevant prognostic significance.

Notably, grade 3 histology was among the determinants of high-risk classification in the expanded Oncotype DX/RSClin N + model, where it was associated with a marked increase in the absolute benefit of chemotherapy.^47^ This parallels the GIM2 composite measure of recurrence risk (CPRS) analysis, in which grade 3 contributed to the highest-risk quartiles, characterized by a number needed to treat with dose-dense epirubicin–cyclophosphamide of only 5 and 6, compared with 154 in the lowest-risk quartile.^48^

Taken together, our results suggest that histologic grade should be integrated alongside genomic assays and stage to refine risk stratification and guide appropriate treatment escalation or de-escalation in HR+/HER2– early breast cancer.

Regarding women with HER2+ BC, we observed similar 10-year NS for women with stage I disease (95%), irrespective of HR status. In patients with stage II and III disease, however, the HR+/HER2+ subtype showed more favorable outcomes, with a 4% higher difference in 10-year NS compared to HR−/HER2+ tumors. These patterns are consistent with findings from the Norwegian study by Johansson et al.^28^ Notably, when comparing survival trends across the two time periods (2004-2009 vs. 2010-2014), the greatest improvement in 10-year NS was observed among stage II and III HR−/HER2+ patients, exceeding that of HR+/HER2+ patients at the same stages. These results likely reflect the introduction of the anti-HER2 therapy, namely trastuzumab, which was approved in Italy in 2006 for the treatment of HER2+ non-metastatic BC. Our findings support the hypothesis, at a population level, that trastuzumab may be relatively less effective in HR+/HER2+ disease, particularly in stage II-III, compared to HR−/HER2+ tumors. This is in line with results from a Korean population-based study, which found that trastuzumab did not improve overall survival in patients with triple-positive BC.^49^

The increase in long-term BC survivors observed over the last few decades is largely attributable to advances in diagnostic precision and evolving treatment strategies.^50^

In particular, the consistently high long-term NS among patients with stage I disease reflects the success of early detection efforts. Organized mammography screening programmes,^51^^,^^52^ improved diagnostic tools and technological innovations that enable earlier diagnosis and more accurate staging, have all contributed to this trend. Additionally, treatment efficacy has improved thanks to progress in surgical techniques and the increasing ability to tailor therapies based on individual patient characteristics.^53^ Our findings are consistent with recent published population-based modeling studies showing that survival gains and BC-specific mortality reduction over the past two decades are largely attributable to improvements in systemic therapies for stage II–III disease and metastatic recurrences.^54–56^ These studies support the importance of analyzing stage and subtype jointly to better understand survival dynamics and optimize treatment stratification.

### Strengths and limitations

Our study has several strengths. First, the robustness of the 10-year estimates of NS is supported by the high quality of data from the Friuli Venezia Giulia Cancer Registry^25^ and the availability of long-term, complete follow-up. Second, being population-based, the study reflects outcomes among unselected Italian women with BC, treated according to standard care pathways; notably, our survival estimates are consistent with those reported for Italian patients in other national datasets.^24^ Third, we applied the Pohar-Perme estimator, a well-established method that provides unbiased estimates of cancer-specific (net) survival.^25^^,^^57^

Some limitations of our study should be acknowledged. The cancer registry does not collect information on routine treatment or recurrences,^58^^,^^59^ lifestyle factors (eg, diet, smoking, physical activity), or socioeconomic status, which are known to influence long-term outcomes and could act as unmeasured confounders. However, treatment choices, while continuously evolving, are largely guided by stage and molecular subtype, which are accounted for in our analysis. Nonetheless, the study by Johansson et al^28^ reported that survival estimates remained largely unchanged after adjusting for treatment, suggesting that the absence of treatment data may have a limited impact on our findings. Similarly, while individual lifestyle, socioeconomic data, and individual screening history was not available in our cancer registry database, as well as in most population base registries, on the other hand, the completeness, accuracy, and representativeness of the cancer registries’ data on incidence and survival (eg, follow-up) in our region represent an important strength of the study. In addition, evidence from previous studies^52^^,^^60^ suggest that mammography screening predominantly induces a stage shift, significantly reducing the incidence of advanced-stage breast cancers. Therefore, evaluating 10-year NS within specific stage strata ensures that the observed trends reflect prognostic differences and therapeutic advancements rather than an artificial prolongation of survival time.

Another drawback is the relatively small population size covered by our cancer registry, which limited the statistical power to investigate long-term outcomes for certain important combinations of tumor characteristics. In particular, we were unable to analyze survival trends by age at diagnosis in conjunction with both stage and molecular subtype: classifying patients across multiple categories—age, stage, and subtype—simultaneously results in very small subgroups of patients for which long-term estimation of survival were statistically unstable and poorly comparable over time and areas.

However, when stratifying by subtype only, our results showed that HR+/HER2– patients diagnosed before age 40 had poorer survival than women in the perimenopausal and screening age groups. This finding suggests that younger age at diagnosis is a particularly adverse prognostic factor in HR+/HER2– BC, as previously highlighted.^61^^,^^62^

Survival estimates in our study were derived using conventional cohort-based analysis, which requires long-term follow-up and may reflect treatment practices that are no longer current. While more recent or future survival projections can be obtained through alternative approaches, such as period analysis^63^ or mixture cure models^64^ these methods rely on assumptions that may not always be verifiable.

### Clinical relevance

Our results revealed that additional information on survival outcomes was obtained when prognostic factors were examined in combination. Within each stage, significant variation in survival was seen due to molecular subtypes, age or histologic grade. These findings may provide important information to oncologists for planning follow-up and may have implications for patient-tailored treatment. Moreover, these estimates are highly useful for breast cancer survivors who can benefit from accurate information on their long-term prognosis.

## Conclusions

Cancer registries data with long-term and complete follow-up of patients can play a key role in monitoring how targeted therapies impact BC prognosis in specific patient subgroups. Such studies provide clinicians with real-world evidence on the long-term survival of women with BC by stage, grade, and molecular subtype, thereby supporting the continued refinement and implementation of personalized treatment and follow-up strategies.

## Supplementary Material

oyag338_Supplementary_Data

## Data Availability

The datasets analyzed during the current study are not publicly available due to confidentiality. Further information is available from the corresponding author upon request.
