# Supplementary material for: Ten-year survival of women with invasive breast cancer by stage, molecular subtype, and grade combined: a population-based study
Source: Oncologist. 2026 Aug 25;31(10):oyag338. doi: 10.1093/oncolo/oyag338 (PMC13576421; doi:10.1093/oncolo/oyag338)
Supplement: Supplementary file 1 [file oyag338_supplementary_data.zip › Supplementary Material_Tables.docx]

**Supplementary Tables**

**Journal:The Oncologist**

**Title:Ten-year survival of women with invasive breast cancer by stage, molecular subtype, and grade combined: a population-based study in North-Eastern Italy**

**Fabiola Giudici^1*^, Diego Serraino^1^, Fabio Puglisi^2,3^, Samuele Massarut^4^, Lorenzo Gerratana^2,3^, Serena Scomersi^5^, Federica Toffolutti^1^, Ettore Bidoli^1^, Luigino Dal Maso^1,^ and Friuli Venezia Giulia Cancer Registry working group^6^**

^1^Cancer Epidemiology Unit, Centro di Riferimento Oncologico di Aviano (CRO) IRCCS, Aviano, Italy;

^2^Department of Medical Oncology, Centro di Riferimento Oncologico di Aviano (CRO) IRCCS, Aviano, Italy;

^3^Department of Medicine, University of Udine, Udine, Italy.

^4^Breast Surgery Unit, Centro di Riferimento Oncologico di Aviano (CRO) IRCCS, Aviano, Pordenone, Italy;

^5^ Breast Surgery Unit, Cattinara University Hospital, Trieste, Italy.

^6^ Members of Friuli Venezia Giulia CancerRegistry Working Group: Tiziana Angelin, Emilia De Santis, Ornella Forgiarini, Elodie Lazzaretto, Mariella Losciale, Martina Taborelli (Cancer Epidemiology Unit – CRO Aviano).

***Corresponding author:**

Fabiola Giudici, Cancer Epidemiology Unit, Centro di Riferimento Oncologico di Aviano (CRO) IRCCS, Via Franco Gallini 2, 33081 Aviano (PN), Italy, Tel: (+39) 0434 659354; Fax: (+39) 0434 659231, E-mail: [fabiola.giudici@cro.it](mailto:fabiola.giudici@cro.it) ORCID: 0000-0002-4160-3479

**Table of contents**

**Supplementary Table 1.** Molecular profile and TNM Stage according to age at diagnosis for women with invasive breast cancer, Friuli Venezia Giulia Cancer Registry, 2004-2014, age 20-74 years……………....3

**Supplementary Table 2**. Distribution by Stage at Diagnosis and Molecular Profile (N=8,402, 80.3%), 2004-2014, RT-FVG.......................................................................................................................................4

**Supplementary Table 3.** Net Survival (NS) according to TNM Stage, Molecular profile and period of diagnosis, for women with invasive breast cancer, Friuli Venezia Giulia Cancer Registry, 2004-2014, age 20-74 years …...……………...…………………………………………………………………………..….5

**Supplementary Table 4.** Net Survival (NS) according to HR+/HER2-, HR-/HER2- and Grade, for women with invasive breast cancer, Friuli Venezia Giulia Cancer Registry, 2004-2014, age 20-74 year…………………………………………………………………………………………………………..6

**Supplementary Table 1. Molecular profile and TNM Stage according to age at diagnosis for women with invasive breast cancer, Friuli Venezia Giulia Cancer Registry, 2004-2014, age 20-74 years**

| Variable | N | Overall,  N = 10,467 | Age at diagnosis | | | | |
| --- | --- | --- | --- | --- | --- | --- | --- |
|  |  |  | **20-39**  **N = 536** | **40-49**  **N = 2,105** | **50-59**  **N = 2,586** | **60-69**  **N = 3,631** | **70-74**  **N = 1,609** |
| Stage | **9,582** |  |  |  |  |  |  |
| I |  | 4,821 (50.3%) | 159 (33.6%) | 920  (48.0%) | 1,216 (51.2%) | 1,839 (54.7%) | 687 (47.1%) |
| II |  | 3,116 (32.5%) | 204 (43.1%) | 677  (35.4%) | 743  (31.3%) | 1,020 (30.3%) | 472 (32.4%) |
| III |  | 1,222 (12.8%) | 82  (17.3%) | 247  (12.9%) | 314  (13.2%) | 363  (10.8%) | 216 (14.8%) |
| IV |  | 423  (4.4%) | 28  (5.9%) | 71  (3.7%) | 100  (4.2%) | 141  (4.2%) | 83  (5.7%) |
| Unknown |  | 885 | 63 | 190 | 213 | 268 | 151 |
|  |  |  |  |  |  |  |  |
| Molecular Profile | **8,709** |  |  |  |  |  |  |
| HR+/HER2- |  | 6,536 (75.0%) | 211 (55.4%) | 1,307 (72.8%) | 1,571 (72.4%) | 2,401 (78.9%) | 1,046 (79.2%) |
| HR+/HER2+ |  | 872  (10.0%) | 68  (17.8%) | 221  (12.3%) | 212  (9.8%) | 267  (8.8%) | 104  (7.9%) |
| HR-/HER2+ |  | 478  (5.5%) | 39  (10.2%) | 110  (6.1%) | 145  (6.7%) | 135  (4.4%) | 49  (3.7%) |
| HR-/HER2- |  | 823  (9.5%) | 63  (16.5%) | 157 (8.7%) | 241  (11.1%) | 241  (7.9%) | 121  (9.2%) |
| Unknown |  | 1,758 | 155 | 310 | 417 | 587 | 289 |
|  |  |  |  |  |  |  |  |
| Histologic Grade | **9,357** |  |  |  |  |  |  |
| G1 |  | 1,337 (14.3%) | 33  (7.1%) | 267  (14.2%) | 327  (14.1%) | 512  (15.6%) | 198  (14.1%) |
| G2 |  | 5,022 (53.7%) | 193 (41.6%) | 981  (52.3%) | 1,173 (50.5%) | 1,850 (56.2%) | 825  (58.7%) |
| G3 |  | 2,998 (32.0%) | 238 (51.3%) | 627  (33.4%) | 822  (35.4%) | 928  (28.2%) | 383  (27.2%) |
| Unknown |  | 1,110 | 72 | 230 | 264 | 341 | 203 |

**Supplementary Table 2. Distribution by Stage at Diagnosis and Molecular Profile (N=8,402, 80.3%), 2004-2014, RT-FVG**

| Variable | Stage | | | |
| --- | --- | --- | --- | --- |
|  | **I**  N = 4,223 | **II**  N = 2,761 | **III**  N = 1,098 | **IV**  N = 320 |
| Profile |  |  |  |  |
| HR+/HER2-  (N=6,339) | 3,407 (80.7%) | 1,998 (72.4%) | 747 (68.0%) | 187 (58.4%) |
| HR+/HER2+  (N=834) | 353 (8.4%) | 292 (10.6%) | 142 (12.9%) | 47 (14.7%) |
| HR-/HER2+  (N=448) | 156 (3.7%) | 160 (5.8%) | 93 (8.5%) | 39 (12.2%) |
| HR-/HER2-  (N=781) | 307 (7.3%) | 311 (11.3%) | 116 (10.6%) | 47 (14.7%) |

**Supplementary Table 3. Net Survival (NS) according to TNM Stage, Molecular profile and period of diagnosis, for women with invasive breast cancer, Friuli Venezia Giulia Cancer Registry, 2004-2014, age 20-74 years**

|  |  | Number of Patients  at diagnosis | | Number of patients  at risk at 10 years | | 5-years NS | | 10-years NS | |
| --- | --- | --- | --- | --- | --- | --- | --- | --- | --- |
| Stage | **Molecular**  **Profile** | **2004-2009** | **2010-2014** | **2004-2009** | **2010-2014** | **2004-2009** | **2010-2014** | **2004-2009** | **2010-2014** |
| I  (N=4,223) | **HR+/HER2-** | 1,711 | 1,696 | 1,546 | 1,535 | 99.5% | 100.0% | 98.6% | 99.0% |
|  | **HR+/HER2+** | 224 | 129 | 198 | 114 | 98.8% | 97.7% | 96.1% | 94.3% |
|  | **HR-/HER2+** | 87 | 69 | 77 | 66 | 98.3% | 93.3% | 96.8% | 92.8% |
|  | **HR-/HER2-** | 151 | 156 | 126 | 130 | 93.0% | 94.8% | 89.8% | 91.2% |
|  |  |  |  |  |  |  |  |  |  |
| II  (N=2,761) | **HR+/HER2-** | 1,220 | 778 | 993 | 644 | 95.9% | 88.6% | 88.9% | 90.9% |
|  | **HR+/HER2+** | 194 | 98 | 169 | 80 | 97.9% | 94.0% | 93.2% | 88.8% |
|  | **HR-/HER2+** | 105 | 55 | 78 | 48 | 89.5% | 95.8% | 83.5% | 93.6% |
|  | **HR-/HER2-** | 184 | 127 | 141 | 95 | 81.2% | 81.8% | 82.1% | 81.2% |
|  |  |  |  |  |  |  |  |  |  |
| III  (N=1,098) | **HR+/HER2-** | 426 | 321 | 269 | 190 | 86.7% | 86.0% | 68.4% | 65.0% |
|  | **HR+/HER2+** | 97 | 45 | 59 | 26 | 85.2% | 81.4% | 65.0% | 65.8 % |
|  | **HR-/HER2+** | 61 | 32 | 28 | 25 | 66.9% | 83.4% | 48.6% | 87.9% |
|  | **HR-/HER2-** | 62 | 54 | 16 | 21 | 43.1% | 52.8% | 28.8% | 42.3% |
|  |  |  |  |  |  |  |  |  |  |
| IV  (N=320) | **HR+/HER2-** | 94 | 93 | 7 | 8 | 24.1% | 32.3% | 7.8% | 9.2% |
|  | **HR+/HER2+** | 24 | 23 | 3 | 2 | 17.2% | 31.1% | 13.7% | 9.1% |
|  | **HR-/HER2+** | 19 | 20 | 4 | 3 | 26.9% | 25.3% | 22.9% | 15.2% |
|  | **HR-/HER2-** | 28 | 19 | 1 | 2 | 3.7% | 15.9% | 3.7% | 11.4% |

NS:Net Survival

**Supplementary Table 4. Net Survival (NS) according to HR+/HER2-, HR-/HER2- and Grade, for women with invasive breast cancer, Friuli Venezia Giulia Cancer Registry, 2004-2014, age 20-74 years**

|  |  |  | 5-years | | 10-years | |
| --- | --- | --- | --- | --- | --- | --- |
| Stage | **Grade** | **Patients**  **N (%)** | **Number at risk** | **NS** | **Number at risk** | **NS** |
| HR+/HER2-  (N=6,237) | **G1** | 1,083 (17.4%) | 1,043 | 99.6% | 975 | 98.3% |
|  | **G2** | 3,869 (62.0%) | 3,605 | 96.4% | 3,219 | 90.9% |
|  | **G3** | 1,285 (20.6%) | 1,101 | 88.5% | 946 | 79.9% |
|  |  |  |  |  |  |  |
| HER2+  (N=1,266) | **G1** | 52 (4.1%) | 49 | 97.9% | 47 | 98.1% |
|  | **G2** | 445 (35.2%) | 399 | 92.2% | 350 | 85.1% |
|  | **G3** | 769 (60.7%) | 651 | 87.5% | 580 | 81.9% |
|  |  |  |  |  |  |  |
| HR-/HER2-  (N=753) | **G1** | 19 (2.5%) | 19 | 100.0% | 19 | 100.0% |
|  | **G2** | 133 (17.7%) | 104 | 81.5% | 91 | 75.5% |
|  | **G3** | 601 (79.8%) | 447 | 76.8% | 412 | 74.1% |

NS:Net Survival
