# Supplementary material for: Ten-year survival of women with invasive breast cancer by stage, molecular subtype, and grade combined: a population-based study
Source: Oncologist. 2026 Aug 25;31(10):oyag338. doi: 10.1093/oncolo/oyag338 (PMC13576421; doi:10.1093/oncolo/oyag338)
Supplement: Supplementary file 1 [file oyag338_supplementary_data.zip › Supplementary Material_Figures.docx]

**Supplementary Figures**

**Journal:The Oncologist**

**Title:Ten-year survival of women with invasive breast cancer by stage, molecular subtype, and grade combined: a population-based study in North-Eastern Italy**

**Fabiola Giudici^1*^, Diego Serraino^1^, Fabio Puglisi^2,3^, Samuele Massarut^4^, Lorenzo Gerratana^2,3^, Serena Scomersi^5^, Federica Toffolutti^1^, Ettore Bidoli^1^, Luigino Dal Maso^1,^ and Friuli Venezia Giulia Cancer Registry working group^6^**

^1^Cancer Epidemiology Unit, Centro di Riferimento Oncologico di Aviano (CRO) IRCCS, Aviano, Italy;

^2^Department of Medical Oncology, Centro di Riferimento Oncologico di Aviano (CRO) IRCCS, Aviano, Italy;

^3^Department of Medicine, University of Udine, Udine, Italy.

^4^Breast Surgery Unit, Centro di Riferimento Oncologico di Aviano (CRO) IRCCS, Aviano, Pordenone, Italy;

^5^ Breast Surgery Unit, Cattinara University Hospital, Trieste, Italy.

^6^ Members of Friuli Venezia Giulia CancerRegistry Working Group: Tiziana Angelin, Emilia De Santis, Ornella Forgiarini, Elodie Lazzaretto, Mariella Losciale, Martina Taborelli (Cancer Epidemiology Unit – CRO Aviano).

***Corresponding author:**

Fabiola Giudici, Cancer Epidemiology Unit, Centro di Riferimento Oncologico di Aviano (CRO) IRCCS, Via Franco Gallini 2, 33081 Aviano (PN), Italy, Tel: (+39) 0434 659354; Fax: (+39) 0434 659231, E-mail: [fabiola.giudici@cro.it](mailto:fabiola.giudici@cro.it) ORCID: 0000-0002-4160-3479

**Table of contents**

**Supplementary Figure 1**. Overall and net survival until 10 years after a diagnosis of breast cancer in women. Friuli Venezia Giulia Cancer Registry, Italy, 2004-2014, age 20-74 years………………………..3

**Supplementary Figure 2.** Net Survival (NS) according to TNM Stage and Grade, for women with invasive breast cancer, Friuli Venezia Giulia Cancer Registry, 2004-2014, age 20-74 years.………...……4

**Supplementary Figure 3.** Net survival estimates in different age groups: Overall (A) and by molecular profile (B). Friuli Venezia Giulia Cancer Registry, 2004-2014, age 20-74 years……………………..……5

**Supplementary Figure 4.** Net Survival according to Stage at diagnosis (n=9,582) (A) and according to Molecular profile available (n=8,709) (B), Friuli Venezia Giulia Cancer Registry, 2004-2014, age 20-74 years……….…………………………………………………………………………………………..…… 6

**Supplementary Figure 1. Overall and net survival until 10 years after a diagnosis of breast cancer in women. Friuli Venezia Giulia Cancer Registry, Italy, 2004-2014, age 20-74 years**

**Supplementary Figure 2. Net Survival (NS) according to TNM Stage and Grade, for women with invasive breast cancer, Friuli Venezia Giulia Cancer Registry, 2004-2014, age 20-74 years**

|  |  |  | 5-years | | 10-years | |
| --- | --- | --- | --- | --- | --- | --- |
| Stage | **Grade** | **Patients**  **N (%)** | **Number at risk** | **NS** | **Number at risk** | **NS** |
| I  (N=4,665) | **G1** | 1,004 (21.5%) | 967 | 99.7% | 916 | 99.7% |
|  | **G2** | 2,650 (56.8%) | 2,544 | 99.4% | 2,358 | 97.4% |
|  | **G3** | 1,011 (21.7%) | 962 | 97.8% | 897 | 95.5% |
|  |  |  |  |  |  |  |
| II  (N=2,997) | **G1** | 252 (8.4%) | 244 | 99.4% | 223 | 96.2% |
|  | **G2** | 1,598 (53.3%) | 1,496 | 96.7% | 1,328 | 90.5% |
|  | **G3** | 1,147 (38.3%) | 998 | 90.0% | 895 | 84.7% |
|  |  |  |  |  |  |  |
| III  (N=1,180) | **G1** | 44 (3.7%) | 40 | 93.4% | 30 | 79.0% |
|  | **G2** | 549 (46.5%) | 470 | 88.2% | 345 | 68.1% |
|  | **G3** | 587 (49.7%) | 419 | 74.2% | 309 | 57.7% |

NS=Net Survival

**Supplementary Figure 3. Net survival estimates in different age groups: Overall (A) and by molecular profile (B). Friuli Venezia Giulia Cancer Registry, 2004-2014, age 20-74 years**

**A)**

**B)**

|  | **Number at risk at 0 and 10 years** | | | | **10-years Net Survival** | | | |
| --- | --- | --- | --- | --- | --- | --- | --- | --- |
|  | **HR+/HER2-** | **HR+/HER2+** | **HR-/HER2+** | **HR-/HER2-** | **HR+/HER2-** | **HR+/HER2+** | **HR-/HER2+** | **HR-/HER2-** |
| **Age at diagnosis** |  |  |  |  |  |  |  |  |
| <39 | 211, 166 | 68, 56 | 39, 28 | 63, 47 | 81.8% | 82.9% | 82.1% | 76.3% |
| 40-49 | 1307, 1167 | 221,191 | 110,79 | 157, 115 | 92.5% | 90.4% | 74.4% | 76.8% |
| 50-59 | 1571, 1353 | 212,176 | 145, 113 | 241, 167 | 90.0% | 87.5% | 81.4% | 72.2% |
| 60-69 | 2401, 1956 | 267,192 | 135, 95 | 267, 167 | 90.4% | 79.5% | 78.4% | 76.1% |
| 70-74 | 1046, 686 | 104, 61 | 49, 31 | 104, 61 | 81.5% | 72.8% | 80.0% | 63.8% |

**Supplementary Figure 4. Net Survival according to Stage at diagnosis (n=9,582) (A) and according to Molecular profile available (n=8,709) (B), Friuli Venezia Giulia Cancer Registry, 2004-2014, age 20-74 years**

| N=8,709 | 5-year NS | 10-year NS |
| --- | --- | --- |
| HR+/HER2- (n=6,536) | 94.7% | 89.0% |
| HR+/HER2+ (n=872) | 90.9 % | 83.6% |
| HR-/HER2+ (n=478) | 83.8% | 78.8% |
| HR-/HER2- (n=823) | 76.6% | 73.2% |
| NS: Net survival |  |  |

| N=9,582 | 5-year NS | 10-year NS |
| --- | --- | --- |
| Stage I (n=4,821) | 99.1% | 97.5% |
| Stage II (N=3,116) | 94.3% | 88.5% |
| Stage III(N=1,222) | 81.1% | 62.9% |
| Stage IV (N=423) | 22.4% | 9.1% |
| NS: Net survival |  |  |

**A)	by stage	B)	by molecular profile**
