# Supplementary material for: Ten-year survival of women with invasive breast cancer by stage, molecular subtype, and grade combined: a population-based study
Source: Oncologist. 2026 Aug 25;31(10):oyag338. doi: 10.1093/oncolo/oyag338 (PMC13576421; doi:10.1093/oncolo/oyag338)
Supplement: Supplementary file 1 [file oyag338_supplementary_data.zip › SUPPLEMENTARY MATERIAL_R Code.docx]

**Supplementary Material**

**Journal:The Oncologist**

**Title:Ten-year survival of women with invasive breast cancer by stage, molecular subtype, and grade combined: a population-based study in North-Eastern Italy**

**Fabiola Giudici^1*^, Diego Serraino^1^, Fabio Puglisi^2,3^, Samuele Massarut^4^, Lorenzo Gerratana^2,3^, Serena Scomersi^5^, Federica Toffolutti^1^, Ettore Bidoli^1^, Luigino Dal Maso^1,^ and Friuli Venezia Giulia Cancer Registry working group^6^**

^1^Cancer Epidemiology Unit, Centro di Riferimento Oncologico di Aviano (CRO) IRCCS, Aviano, Italy;

^2^Department of Medical Oncology, Centro di Riferimento Oncologico di Aviano (CRO) IRCCS, Aviano, Italy;

^3^Department of Medicine, University of Udine, Udine, Italy.

^4^Breast Surgery Unit, Centro di Riferimento Oncologico di Aviano (CRO) IRCCS, Aviano, Pordenone, Italy;

^5^ Breast Surgery Unit, Cattinara University Hospital, Trieste, Italy.

^6^ Members of Friuli Venezia Giulia CancerRegistry Working Group: Tiziana Angelin, Emilia De Santis, Ornella Forgiarini, Elodie Lazzaretto, Mariella Losciale, Martina Taborelli (Cancer Epidemiology Unit – CRO Aviano).

```
################################################################################
# SUPPLEMENTARY MATERIAL: R Script for Relative Survival Analysis
# Using the 'relsurv' package
################################################################################
=========================================================================
# STEP 1: Setting up the Environment and Importing Population Life Tables
=========================================================================
#  LOAD REQUIRED PACKAGES
# Ensure you have installed 'survival' and 'relsurv' before running
# install.packages(c("survival", "relsurv"))
library(survival)
library(relsurv)

#  SET UP THE POPULATION LIFE TABLES (Ratetable)
# NOTE: this code is for Italian tables and must be adapted according the life tables of your country (available at https://www.mortality.org/)
#import in txt format life tables for males and females: the two files contain three variables: 'Year' (calendar year), 'Age' (individual age), and 'qx' (probability of death).
```

#the directory in R must be the same that life tables files

read.table("your directory/.. female.txt", header=TRUE,stringsAsFactors=TRUE, sep="", na.strings="NA", dec=".", strip.white=TRUE)

read.table("your directory/…….male.txt", header=TRUE,stringsAsFactors=TRUE, sep="", na.strings="NA", dec=".", strip.white=TRUE)

\# Convert life tables to the format required by 'relsurv'

\# Replace 'fvgpop' with your local population rate table (e.g., US, UK..)

#To calculate relative survival, background population mortality rates stratified by age and calendar year are required. The 'relsurv' package processes this population data into a structured 'transrate' object.

\# This function formats raw life table data into the required rate object.

\# fvgpop <- transrate.hmd(male = "male.txt",female = "female.txt")

#Check the structure the created object

\# head(fvgpop)

This command performs a visual quality check on the final rate table object. The output displays the top rows of the multi-dimensional array, confirming that the object is properly stratified by 'age', 'year', and 'sex'. This step ensures that the original probability of death (qx) values life tables have been successfully converted into the continuous mortality rates required by the relative survival models

```
=========================================================================
# STEP 2: Estimating Net Survival using the Pohar-Perme Method
=========================================================================
#  DATA PREPARATION
# Replace 'breast_fvg_data' with your actual dataset object name: this is the file with clinical and survival data of your population
# NOTE: The 'relsurv' package requires follow-up time to be strictly in DAYS.
# Age must also be converted to days when mapped to the rate table.
# The year of cancer diagnosis (diag) must be specified in days (starting from 01/01/1960).

# data <- breast_fvg_data
# data$time <- data$survival_time_years * 365.241
# data$age  <- data$age_years * 365.241

# Calculate the net survival using the 'rs.surv' function

fit_rs <- rs.surv(
formula   = Surv(time, status) ~ 1,            # Add covariates (e.g. ~ stage)
data = data,                                   # Replace with your dataset
ratetable = fvgpop,                            # Replace with your life table
rmap = list(age = age, sex = sex, year = diag) # Mapping list
method    = "pohar-perme"                      # Gold standard for NS
add.times = c(5, 10) * 365.241,                                          # Compute NS at 5 and 10 years
)
# NOTE: rmap = list(...): This mapping argument is crucial. It aligns the individual patient characteristics from the clinical dataset (data) with the corresponding dimensions of the population life tables (fvgpop). Specifically, it maps the patient's age at diagnosis (age), sex (sex), and exact calendar year of diagnosis (diag) to ensure the correct baseline expected mortality is subtracted.


# Plot the relative survival curve
plot(fit_ns, xlab = "Time (Years)", ylab = "Survival",
     main = "All Cohort", col = "blue", lwd = 2)
# Example: Supplementary Figure 1(Plot both Overall and Net Survival)
overall_surv <- survfit(Surv(survival_time_years, status)~1, data = data)
plot(overall_surv, xlab = "Time (years)", ylab = "Survival",  yscale=100,col=c("darkblue"),main="All Cohort",ylim=c(0.0,1),xlim=c(0,10),conf.int=FALSE);par(new=TRUE)
fit_net <- rs.surv(Surv(time, status) ~ 1, data = data, ratetable = fvgpop, method = "pohar-perme", add.times = c(5, 10) * 365.241, rmap = list(age = age, sex = sex, year = diag))
plot(fit_net, xlab = "Time (years)",  yscale=100,xscale = 365.241,col=c("red"),ylim=c(0.0,1),conf.int=FALSE)
legend("bottomleft",fill=c("blue","red"),legend=c("Overall Survival","Net Survival"),bty="n")
################################################################################
# END OF SCRIPT
################################################################################
```
